# Prevalence and risk factors associated with malaria infection among pregnant women in a semi-urban community of north-western Nigeria

**DOI:** 10.1186/s40249-015-0054-0

**Published:** 2015-04-24

**Authors:** Sani Abdullahi Fana, Mohammed Danladi Abubakar Bunza, Sule Aliyu Anka, Asiya Umar Imam, Shehu Usman Nataala

**Affiliations:** Department of Medical Microbiology, Faculty of Medical Laboratory Science, UsmanuDanfodiyo University, Sokoto, Nigeria; Department of Biological Sciences, UsmanuDanfodiyo University, Sokoto, Nigeria

**Keywords:** Malaria, Pregnant women, Mean parasite density, Prevalence, Risk factors, Giemsa stain, Argungu

## Abstract

**Background:**

Malaria during pregnancy remains a serious public health problem, with substantial risks for the mother, her foetus and the newborn. The aim of this study was to determine the prevalence of malaria and possible risk factors for malaria infection among pregnant women in a semi-urban area in north-western Nigeria. Pregnant women are among the most susceptible to malaria infection. Knowledge of their malaria infection status is an important yardstick to measure the effectiveness of any malaria control programme.

**Methods:**

We conducted a cross sectional study in the semi-urban area of Argungu, Kebbi State Nigeria. Two hundred and fifty five pregnant women were included in the study after informed verbal consent was obtained. For each participant, the socio-demographic profile, stage of pregnancy and attitude to the use of insecticide- treated nets (ITNs) were investigated using a questionnaire. Peripheral blood samples were collected and thick blood smears were prepared and stained with Giemsa stains to check for malaria parasitaemia. The associations between age, education level and use of ITNs with occurrence of malaria infection during pregnancy were analysed using the chi-square test.

**Results:**

One hundred and six (41.6%) out of 255 pregnant women were infected with malaria parasites, with a mean parasite density of 800 parasitesμl^−1^. It was found that prevalence and parasite density decreased as age increased. The chi-square test indicated that a lack of education and non-usage of ITNs were significantly associated with malaria infection.

**Conclusion:**

Malaria is still a major public health issue among pregnant women mainly due to illiteracy and non -compliance to using ITNs. Increasing awareness about malaria preventive measures and early attendance of antenatal care services will help to reduce malaria and, consequently, its associated morbidities and mortalities.

**Electronic supplementary material:**

The online version of this article (doi:10.1186/s40249-015-0054-0) contains supplementary material, which is available to authorized users.

## Multilingual abstracts

Please see Additional file 1 for translations of the abstract into the six official working languages of the United Nations.

## Background

Malaria is a life threatening parasitic disease caused by the protozoa of the genus *Plasmodium.* Five species are known to inflict humans namely, *P. falciparum, P. malaria, P. ovale, P. vivax,* and *P. knowlesi.* The disease is transmitted by the bite of infected female *Anopheles* mosquitoes. Malaria in pregnancy is caused mainly by the specie *P. falciparum*, which is the most common species in Africa.In malaria endemic regions, individuals are constantly exposed to malaria parasites through bites of the aforementioned mosquitoes. This frequent exposure leads to the development of an effective anti-disease immunity, which prevents life-threatening parasite burdens and suppresses the pro-inflammatory responses that cause illness [1] Malaria infection during pregnancy is a major public health problem- especially in tropical and sub-tropical regions; with substantial risks for the mother, her foetus and the newborn. Most cases of malaria in pregnancy in areas of stable malaria transmission are asymptomatic [2]. Depending on the endemicity of malaria in an area, it can be expected that 1–50% of pregnant women may carry malaria parasitaemia, especially in the placenta, without noticing it [3]. This is attributed to anti-disease immunity acquired during previous exposure that protects against clinical malaria [4]. Pregnant women are three times more likely to suffer from severe diseases as a result of malarial infection compared with their non-pregnant counterparts, and have a mortality rate that approaches 50% [5]. The principal impact of malaria infection is due to the presence of parasites in the placenta, which causes maternal anaemia and low birth weight [6]. Beyond the post-partum period, the long term consequences of malaria during pregnancy on the infant include poor development, behavioural problems, short stature and neurological deficits [7].

Protection of pregnant women living in malaria endemic countries has been of particular interest to many malaria control programmes because of this group’s higher susceptibility and reduced immunity. Nigeria, accounts for one fourth of all malaria cases in the 45 endemic countries in Africa [8], and 11% of maternal deaths in the country are attributed to malaria [9]. Positively, malaria control measures have received a greater attention in the last decade as increased funding has resulted in the scaling up of malaria control programmes. Use of insecticide-treated nets (ITNs) is one of the key components of malaria prevention and control as recommended by the World Health Organization (WHO) [10]. The nets reduce human contact with mosquitoes, thus leading to a significant reduction in the incidence of malaria, associated morbidity, and mortality; as well as in the adverse effects during pregnancy in areas of intense malaria transmission [11].

Another key intervention for controlling malaria and its effects during pregnancy is the administration of intermittent preventive treatment (IPT). This consists of a full therapeutic course of antimalarial medicine given to pregnant women at routine prenatal visits, regardless of whether they are infected with malaria or not. Intermittent preventive treatment reduces incidences of maternal malaria episodes, maternal and foetal anaemia, placental parasitaemia, low birth weight and neonatal mortality. Therefore, the WHO recommends IPT with sulfadoxine-pyrimethamine in areas with moderate to high malaria transmission in Africa [12].

As the Roll Back Malaria (RBM) programme has been around for more than a decade in Africa, it is time to assess the current impact of the disease among pregnant women who are considered to be one of the most vulnerable groups. This forms the basis for the present study, which aimed at assessing the prevalence of malaria infection and associated risk factors among pregnant women in a semi-urban area of north-western Nigeria.

## Methods

### Study site and study design

A cross-sectional survey was undertaken from August to October 2012 at the Kokani south ward located in the semi-urban area of Argungu, north- west Nigeria.

### Data collection

#### Sample size

The sample size calculation was based on the formula described by Araoye [13] for estimating sample size. We used a malaria prevalence of 22.29% from a previous study [14] at a 95% confidence interval (CI) and a 5% margin of error. A sample size of 266 pregnant women was required. Only 255 participated, with 11 declining due to their husbands not allowing them to participate.

#### Data collection

A census to determine the number of households with pregnant women was conducted prior to sampling. A total number of 850 households were listed to form the sampling frame. Out of this 266 pregnant women in their second trimester were randomly selected based on interviews with household heads. Trained research assistants administered the questionnaires. Data on the age, education level and ITNs usages of the selected pregnant women were collected and sorted.

#### Laboratory methods

One ml of peripheral blood samples were collected by three medical laboratory scientists through veni-puncture from all recruited pregnant women and later taken to the laboratory at the General Hospital Argungu for detection of malaria parasites. Thick blood films were prepared on the grease- free, clean glass slides by spreading two drops of blood over a diameter of 15 mm. The slides were made in duplicates for each subject and labelled accordingly, and were allowed to dry for 24–48 hours before being stained with Giemsa at pH 7.2 for 45 minutes. Two medical laboratory scientists examined the stained slides and discrepancies between the two scientists were resolved by re-examination before the final result was determined. A slide was considered negative when 100 high- power fields were examined under oil immersion objective. Taking the number of leucocytes per micro-litre of blood as 6,000, parasite density was expressed as: parasite count × 6,000 divided by the number of leucocytes counted as described elsewhere [15].

#### Statistical analysis

Data generated from the study were analysed using IBM SPSS statistical software version 20. Chi-square test was used to study associations and differences.

### Ethical consideration

Ethical approval for the study was obtained from the Ethical Committee of the Ministry of Health in Kebbi State. Verbal informed consent from all the participating pregnant women was also obtained. They participants were informed that participation in the research was voluntary and they could opt out at any stage of the research. Pregnant women who were diagnosed with malaria infection were treated using artemisinin-based combination therapies (ACTs) according to national malaria treatment policy in Nigeria.

## Results

The demographic characteristics of the 255 pregnant women who participated in this study are summarised in Table 1. The average age of the participants was 26.1 ± 1.7 years (range:- 14 – 41). Over one- third (36.1%) had no education, whilst 25.0%, 21.6%, and 17.3% had primary, secondary and tertiary education, respectively. One hundred and forty-eight women used ITNs (58.0%), while 107 (42.0%) didn’t use nets. Malaria prevalence was 41.6%. Age was not significantly associated with malaria prevalence (*x*^2^ = 5.27, p = 0.153); the 14 – 20 age group had the highest prevalence (51.6%), as well as highest mean parasite density (800 parasitesμl^−1^ of blood) (see Table 2).

**Table 1:** **Demographic characteristics of pregnant women in the study area**

**Demographic**	**No. (%)**
Age group (years)	
14 - 20	62(24.3)
21 - 27	83(32.6)
28 - 34	74(29.0)
35 - 41	36(14.1)
Education level	
None	92(36.1)
Primary	64(25.0)
Secondary	55(21.6)
Tertiary	44(17.3)
Usage of ITNs	
Yes	148(58.0)
No	107(42.0)

**Table 2:** **Prevalence of malaria parasites and mean parasite density of pregnant women in the study area, by age**

**Age(years)**	**No. examined**	**No. infected**	**% infected**	**MPD** ^*^ **(no.pμl** ^**−1**^ **)** ^**¶**^
14 - 20	62	32	51.6	800
21 - 27	83	36	43.4	760
28 - 34	74	27	36.5	640
35 - 41	36	11	30.6	600
Total	255	106	41.6	800

*Mean parasite density (arithmetic mean); ¶: Number of parasitesμl^−1^.

There was a significant association between malaria prevalence and education (*x*^2^ = 20.9, p = 0.000). Malaria prevalence in women with no education was 63.0%, while in those with primary, secondary and tertiary education, it was 45.3%, 32.7%, and 27.3% respectively (see Table 3).

**Table 3:** **Prevalence of malaria parasites and mean parasite density of pregnant women in the study area, by education level**

**Education level**	**No. examined**	**No. infected**	**% infected**	**MPD(no.pμl** ^**−1**^ **)**
None	92	58	63.0	800
Primary	64	29	45.3	740
Secondary	55	18	32.7	640
Tertiary	44	12	27.3	600
Total	255	106	41.6	800

Use of ITNs was significantly associated with malaria prevalence and parasite density, as the number of participants who did not use ITNs regularly reported a high occurrence of malaria infection with a high parasite density, as compared to those who used ITNs on a daily basis (*x*^2^ = 33.6, p = 0.000). While malaria prevalence and parasite density were 62.6% and 800 parasitesμl^−1^ of blood among non- ITN users, it was 26.4% and 600 parasitesμl^−1^ of blood, respectively, among ITN users (see Table 4).

**Table 4:** **Prevalence of malaria parasites and mean parasite density of pregnant women in the study area, by usage of ITNs**

**Usage of ITNs**	**No. examined**	**No. infected**	**% infected**	**MPD(no.pμl** ^**−1**^ **)**
Yes	148	39	26.4	600
No	107	67	62.6	800
Total	255	106	41.6	800

## Discussion

In this study, the prevalence of malaria infection among pregnant women in Argungu was found to be 41.6%. This finding is higher than in Maiduguri where a prevalence of 22.1% was reported among pregnant women [16]. It also contrasts sharply with findings in Lagos, where a prevalence rate of 7.7% among pregnant women attending antenatal clinics for the first time during current pregnancy was reported [17]. However, these findings corroborated with the results in Otukpo, Benue State, where a total prevalence of 42.3% was recorded [18]. This high rate of malaria among pregnant women in the area urgently calls for the need to review the control measures available, with a view to possibly redesigning the control programmes.

In our study, it was observed that maternal age was associated with malaria prevalence, showing that a pregnant woman of younger maternal age is at a greatest risk of malaria infection, as well as having the highest parasite densities. Similar findings have been reported in Lagos where prevalence and parasite density were observed to decrease as age increased [19] It has been consistently demonstrated that infection rates are higher in women in their first and second pregnancies, with lower rates in later pregnancies [20]. This is understandable as pregnancy is naturally accompanied by general immune suppression that may cause loss of acquired immunity to malaria especially among primigravidae. This is because they lack the specific immunity to placental malaria that is acquired from exposure to malaria parasites during pregnancy [4]. This immunity accumulates with successive pregnancies, provided there is exposure to malaria infection [21].

There was a strong association between education level and malaria infection. Prevalence of malaria and parasite density among pregnant women in the area decreased proportionately with the increase in education level. It was observed that non- educated pregnant women had the highest prevalence rate, while those with a tertiary level of education had the lowest. However, a previous study conducted in Lagos indicated that education was not significantly associated with malaria infection among pregnant women [19]. This stresses the role education could have on the overall success of malaria control programmes in the region. Government policies should be geared towards improving citizens’ of education statuses in order to reduce the burden of the disease in the country, especially among the most vulnerable population.

The use of ITNs decreases both the number of malaria cases and malaria deaths in pregnant women [22]. In our study, use of ITNs was found to be associated with malaria infection; pregnant women who did not use ITNs frequently, were more affected by malaria as compared to those who did. A previous study conducted in Otukpo also indicated that the rate of malaria increases with a proportionate decrease in the use of ITNs [18].

### Study limitations

Although the study offers some important findings, it also has limitations: The study was cross sectional and the sample size was not very large, therefore the possibility of sampling error cannot be overruled. Factors such as gravidity, trimester, whether IPT was given and frequency of antenatal care visits were not assessed. Also, the study used ITNs as the sole indicator of control measures, but the usage of other measures such as indoor residual spraying (IRS), larvicides and mosquito repellent coils was not assessed.

## Conclusion

Malaria is still a major public health problem among pregnant women in Argungu. Lack of education and non-usage of ITNs were the major factors associated with an increased risk of malaria infection. The control measures available in the area should be reviewed and emphasis should be placed on adequate sensitisation on usage of ITNs. Early attendance and participation in focused ante-natal care services should be encouraged among all pregnant women especially the primigravidae, in order to reduce the risk of malaria infection in pregnancy. Again, awareness on malaria prevention measures during pregnancy should target young women even before marriage preferably at schools, and social and religious gatherings.

## Additional file

Additional file 1:
**Multilingual abstracts in the six official working languages of the United Nations.** (PDF 282 kb)

## Abbreviations

ACTs: Artemisinin-based combination therapies
HIV: Human immunodeficiency virus
IRS: Indoor residual spraying
ITN: Insecticide-treated net
ml: Millilitre
mm: Millimetre
MPD: Mean parasite density
P: Plasmodium
SPSS: Statistical Package for Social Sciences
μl: Microlitre
>: Greater than
<: Less than
%: Percent

## References

[1] Riley EM, Hviid L, Theander TG, Kierszenbaum F (1994). Malaria. Parasitic Infections and the Immune System.

[2] Mockenhaupt FB, Ulmen U, von Gaertner C, Bedu-Addo G, Bienzle U (2002). Diagnosis of placental malaria. J Clin Microbiol.

[3] Steketee RW, Nahlen BL, Parise ME, Menendez C (2001). The burden of malaria in pregnancy in malaria-endemic areas. Am J Trop Med Hyg.

[4] Staalsoe T, Shulman CE, Buhner JN, Kawuondo K, Marsh K, Hviid L (2004). Variant surface antigen-specific IgG and protection against clinical consequences of pregnancy-associated *Plasmodium falciparum* malaria. Lancet.

[5] World Health Organization (2006). Guidelines for the Treatment of malaria.

[6] Rogerson SJ, Boeuf P (2007). New approaches to malaria in pregnancy. Parasitol.

[7] Desai M, terKuile FO, Nosten F, McGready R, Asamoa K, Brabin B, Newman RD (2007). Epidemiology and burden of malaria in pregnancy. Lancet Infect Dis.

[8] World Health Organization (2012). World Malaria Report.

[9] Federal Ministry of Health (2000). Malaria situation analysis document.

[10] World Health Organization (2007). World Malaria Report.

[11] TerKuile FO, Terlouw DJ, Philips-Howard PA, Hawley WA, Friedman JF, Kariuki SK (2003). Reduction of malaria during pregnancy by permethrin-treated bed nets in an area of intense perennial malaria transmission in western Kenya. Am J Trop Med Hyg.

[12] World Health Organization (2004). A strategic framework for malaria prevention and control during pregnancy in the African region.

[13] Araoye MO (2004). Sample Size Determination in Research Methodology with Statistics for Health and Social Sciences 1^st^ ed.

[14] Abdullahi K, Abubakar U, Adamu T, Daneji A, Aliyu RU, Jiya N (2009). Malaria in Sokoto North Western Nigeria. Afri J Biotechnol.

[15] Adefioye OA, Adeyeba OA, Hassan WO, Oyeniran OA (2007). Prevalence of Malaria Parasite Infection among Pregnant women in Osogbo Southwest, Nigeria. American-Eurasian J Sci Res.

[16] Kagu MB, Kawuwa MB, Gadzama GB (2007). Anaemia in Pregnancy: A cross-sectional study of pregnant women in a Sahelian tertiary hospital in Northeastern Nigeria. J Obstet Gynaecol.

[17] Agomo CO, Oyibo WA, Anorlu RI, Agomo PU (2009). Prevalence of Malaria in Pregnant Women in Lagos South-west Nigeria. Kor J Parasitol.

[18] Jombo GTA, Mbaawuaga EM, Ayegba AS, Enenebeaku MNO, Okwori EE, Peters EJ (2010). How far we rolled back malaria on the African continent nine years down? The burden of malaria among pregnant women in a semi-urban community of northern Nigeria. J Med Med Sci.

[19] Agomo CO, Oyibo WA (2013). Factors associated with risk of malaria infection among pregnant women in Lagos Nigeria. Infect Dis Poverty.

[20] Brabin B (1991). An assessment of low birth weight risk in primigravidae as an indicator of malaria control in pregnancy. Int J Epidemiol.

[21] Beeson JG, Duffy PE (2005). The immunology and pathogenesis of malaria during pregnancy. Curr Top Microbiol Immunol.

[22] World Health Organization. Implementation of the Global malaria control strategy. Reportof WHO study Group Geneva: World Health Organization; 1993, General: ISBN9241208392.

