# Supplementary material for: Prevalence and risk factors associated with malaria infection among pregnant women in a semi-urban community of north-western Nigeria
Source: Infect Dis Poverty. 2015 Apr 24;4:24. doi: 10.1186/s40249-015-0054-0 (PMC4534061; doi:10.1186/s40249-015-0054-0)
Supplement: Additional file 1: — Multilingual abstracts in the six official working languages of the United Nations. (PDF 282 kb) [file 40249_2015_54_MOESM1_ESM.pdf]

Translation of the abstract into the six official working languages of the United Nations

أنماط إنتقال داء غابة كياسانور (KFD) في الهند

مانوج مورهيكار، جودادابا قصبي، سانجاي ميهيندال، ديفيندرا ت. موريا، براجيا ياداف و باباصاهب تاندال

الملخص
داء غابة كياسانور (**KFD**) عبارة عن حمى نزفية يسببها فيروس ينتقل بواسطة حشرة القراد وهو داء متوطن في خمس مناطق بولاية كارناتاكا بالهند وتشير التقارير الأخيرة إلى إنتشاره لمناطق مجاورة في غاتس الغربية، وهي مناطق شاماراجانجار في ولاية كارناتاكا ونايلجيري في ولاية تاميل نادو وويناد ومالابورام في ولاية كيرالا وقرية بالي في ولاية غوا، مما يدعو للقلق لذلك فإنه بالإضافة إلى تحصين السكان المصابين، فإن إنشاء نظام مراقبة مبنى على حوادث نفوق القرود في المتنزهات الوطنية ومحميات الحياة البرية والغابات المحمية فى غاتس الغربية سوف يساعد في الكشف عن هذا الداء في وقت مبكر وبالتالى يساعد على إتخاذ تدابير المكافحة المناسبة.

Translated from English version into Arabic by Mohamed R. Habib, through

印度卡萨诺尔森林病的传播模式

Manoj Murhekar, Gudadappa Kasabi, Sanjay Mehendale, Devendra T. Mourya, Pragya Yadav, Babasaheb Tandale

摘要
卡萨诺尔森林病是一种蜱传病毒性出血热，在印度卡纳塔克邦的 5 个区流行。最近有报道称，该病已蔓延至西高止山脉的邻近地区，即卡纳塔克邦查马拉贾纳加尔、泰米尔纳德邦尼尔吉里区、喀拉拉邦瓦亚纳德和默勒布勒姆区、果阿八里村，引起人们的关注。除了给受影响人群进行疫苗接种外，在国家公园、野生动物保护区和西高止山脉的自然森林保护区建立了一个基于猴类死亡的监测系统，用于及时发现疾病，帮助有关部门调整控制措施。

Translated from English version into Chinese by Chen Jin, edited by Yang Pin, through

Schémas de transmission de la fièvre de Kyasanur en Inde

Manoj Murhekar, Gudadappa Kasabi, Sanjay Mehendale, Devendra T. Mourya, Pragya Yadav, Babasaheb Tandale

Résumé
La fièvre de Kyasanur, une fièvre hémorragique transmise par les tiques, est endémique dans cinq districts de l’état

indien de Karnataka. Des signalements inquiétants font état de son apparition récente dans les districts voisins dans les Ghats Occidentaux (districts de Chamarajanagar dans le Karnataka, Nilgiri dans le Tamil Nadu, Wayanad et Malappuram dans le Kerala, village de Pali à Goa). Outre la vaccination de la population menacée, il serait utile de mettre en place un système de surveillance d'événements répertoriant les morts de singes dans les parcs nationaux, les réserves naturelles et les réserves forestières des Ghats Occidentaux, afin de détecter rapidement la maladie et d'aider ainsi à prendre des mesures efficaces pour la combattre.

Translated from English version into French by Suzanne Assenat, through

TRANSLATORS WITHOUT BORDERS

Модели передачи киасанурской лесной болезни (KFD) в Индии

Маной Мурекар, Гудадаппа Казаби, Санджай Мехендале, Девендра Т. Мурия, Прагья Ядав, Бабасахеб Тандале

Реферат

Киасанурская лесная болезнь (KFD), передаваемая клещами вирусная геморрагическая лихорадка, является эндемичной в пяти районах штата Карнатака, Индия. Вызывают беспокойство недавние сообщения о распространении болезни в близлежащих округах в горах Западные Гаты, а именно, в округе Чамараджанагар штата Каматака, в округе Нилгири штата Тамил Наду, в округах Вайанад и Малаппурам штата Керала и в деревне Пали штата Гоа. Кроме вакцинации пораженного населения раннему выявлению случаев заболевания и, следовательно, принятию надлежащих мер контроля помогло бы создание системы наблюдения, основанной на контроле смертности обезьян в национальных парках, заповедниках и заповедных лесах гор Западные Гаты.

Translated from English version into Russian by Alena Hrybouskaya, through

TRANSLATORS WITHOUT BORDERS

Los patrones de transmisión de la enfermedad de la selva de Kyasanur (ESK) en India

Manoj Murhekar, Gudadappa Kasabi, Sanjay Mehendale, Devendra T. Mourya, Pragya Yadav, Babasaheb Tandale

Resumen

La enfermedad de la selva de Kyasanur (ESK), una fiebre hemorrágica viral transmitida por garrapatas, es endémica en cinco distritos del estado de Karnataka, India. Informes recientes de la propagación de la enfermedad a los distritos vecinos en los Ghats occidentales, a saber el distrito Chamarajanagar en Karnataka, el distrito Nilgiri

en Tamil Nadu, los distritos de Wayanad y Malappuram en Kerala, y el poblado de Pali en Goa, son motivo de inquietud. Además de la vacunación de las poblaciones afectadas, el establecimiento de un sistema de monitoreo basado en eventos para la muerte de monos en los parques nacionales, santuarios de vida salvaje y reservas forestales de los Ghats occidentales, ayudaría a detectar la enfermedad de manera precoz y por lo tanto a establecer medidas de control adecuadas.

Translated from English version into Spanish by Maria Alejandra Aguada, through

TRANSLATORS
WITHOUT BORDERS
